# Cutaneous CD30 positive anaplastic large cell lymphoma mimicking breast carcinoma en cuirasse

**DOI:** 10.1016/j.jdcr.2023.07.023

**Published:** 2023-08-19

**Authors:** Patrick McMullan, Regina Brown, Lorin Bibb, Katalin Ferenczi

**Affiliations:** Department of Dermatology, University of Connecticut Health Center, Farmington, Connecticut

**Keywords:** anaplastic large cell lymphoma, brentuximab vedotin, carcinoma en cuirasse, cutaneous lymphoma

## Introduction

Cutaneous anaplastic large cell lymphomas (ALCLs) represent a spectrum of lymphoproliferative disorders, characterized by cutaneous infiltration by neoplastic T cells expressing the transmembrane receptor CD30.^1^^,^^2^ Cutaneous ALCL can present as primary cutaneous disease or as secondary skin manifestation of a systemic-ALCL. It is important to distinguish between these entities as primary cutaneous anaplastic large cell lymphoma (PC-ALCL) and secondary cutaneous ALCL have different clinical course and prognosis^1^, ^2^, ^3^ and patients diagnosed with cutaneous ALCL should therefore undergo workup and staging to assess for systemic involvement. An association has also been established between cutaneous ALCL occurring on the breast in patients with a history of breast implant.^1^ Nonimplant associated cutaneous ALCL of the breast is an extremely rare presentation.^4^^,^^5^ Here we report the case of an 80-year-old woman with systemic-ALCL and no prior history of breast cancer or breast implant, who presented with breast lesions clinically suspicious for “carcinoma en cuirasse.”

## Case report

**Fig 1:**
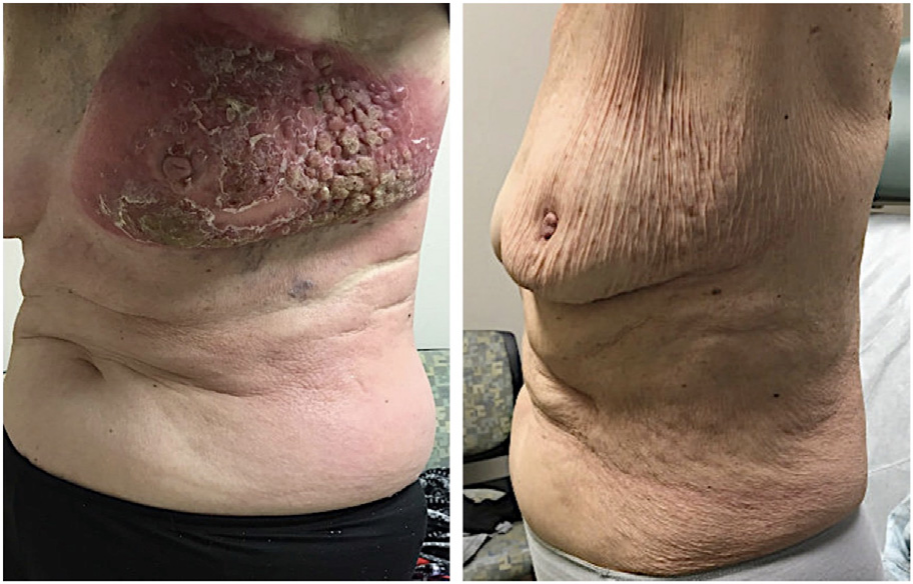
*Left* breast before treatment (*left*) and 5 months following brentuximab vedotin and cyclophosphamide, doxorubicin, and prednisone chemotherapy (*right*).

An 80-year-old woman was referred for a 3-week history of a rapidly expanding, tender, nonpruritic rash with nodules involving the left breast, extending to the left flank. She did not have a history of breast cancer or breast implants and denied systemic symptoms. Cutaneous examination of the left breast revealed a thick, indurated erythematous plaque with overlaying confluent, firm red nodules (Fig 1). Additionally, a red and confluent, indurated nonscaling plaque was appreciated on the left trunk extending to the right trunk. Palpable firm, nonmobile lymphadenopathy was identified in the left axilla.

**Fig 2:**
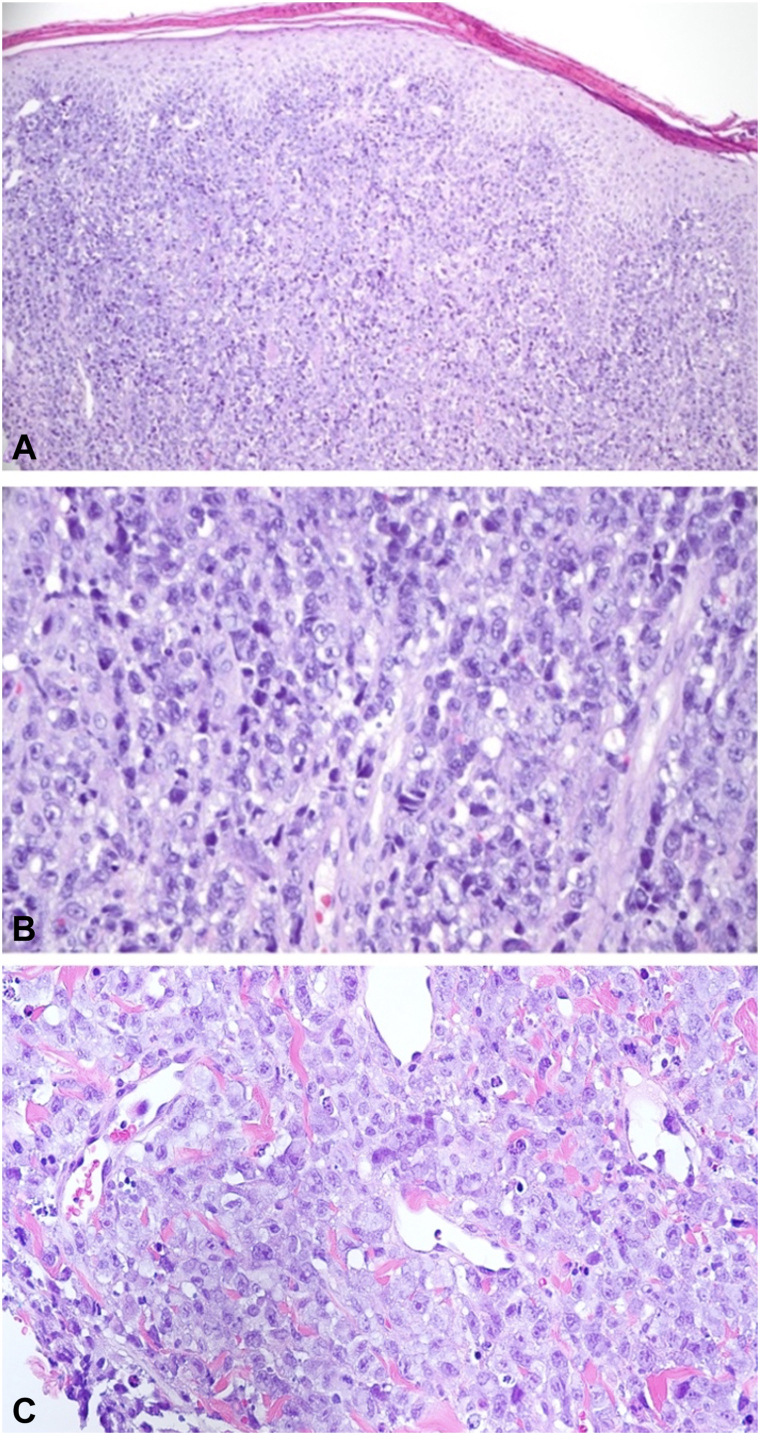
Histologic examination shows: diffuse mononuclear dermal infiltrate (**A**) composed of highly atypical large cells with anaplastic morphology (**B**); hematoxylin and eosin staining: 100× and 400× magnification, respectively. (**C**) Representative hematoxylin and eosin staining demonstrating the presence of atypical large cells within the lymphatic vessel lumen: 400× magnification.

**Fig 3:**
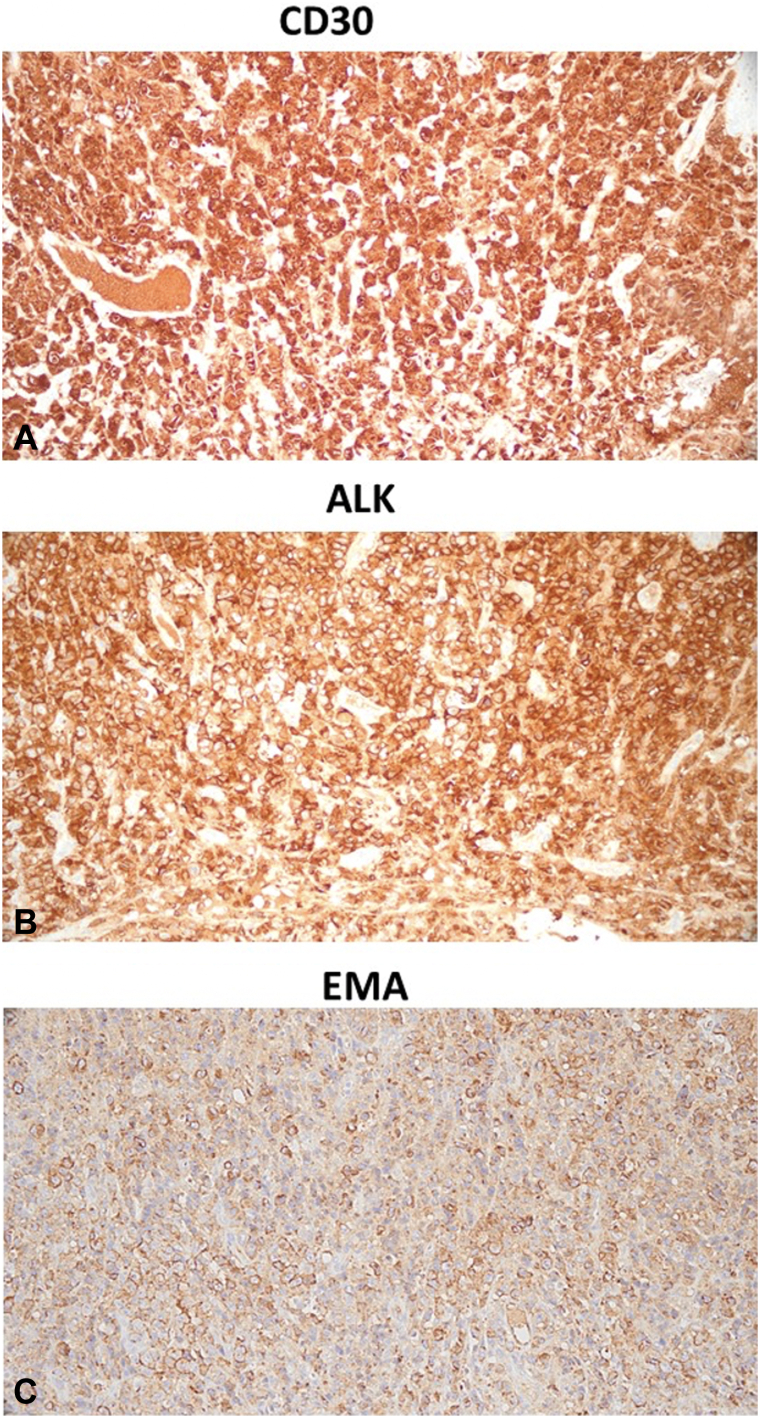
Immunohistochemical staining demonstrating strong and diffuse positive staining for CD30 (**A**), anaplastic lymphoma kinase (**B**), and epithelial membrane antigen (**C**).

**Fig 4:**
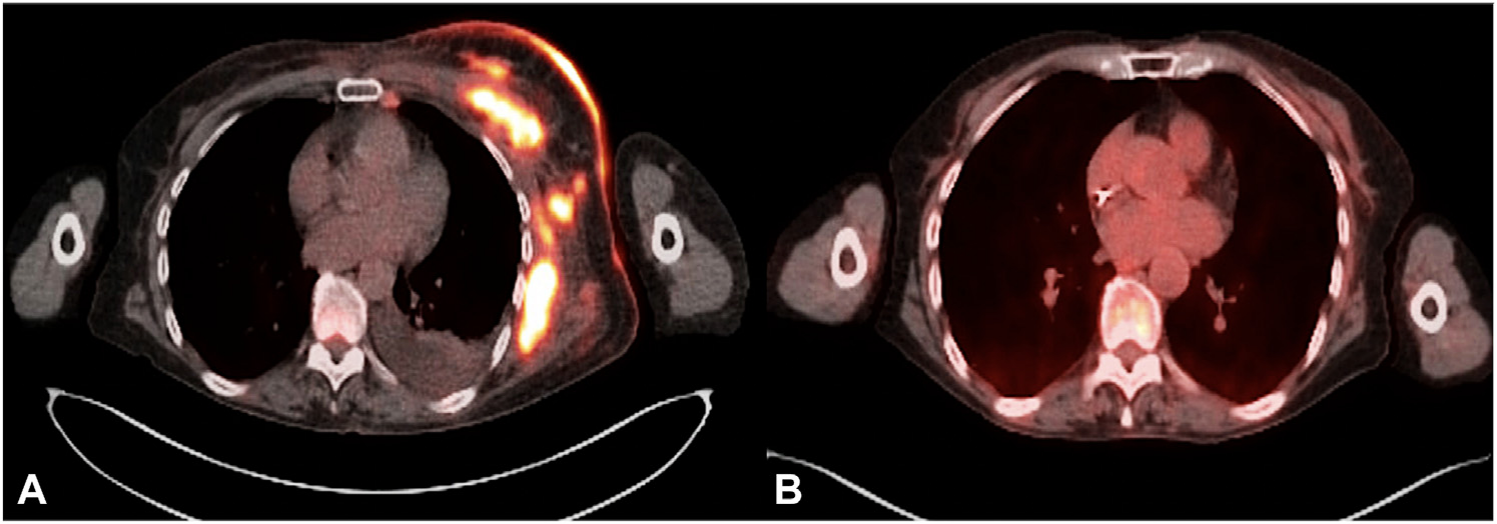
Positron emission tomography/computed tomography scan demonstrating increased fluorine-18 flurodeoxyglucose uptake (*red*) in the *left* breast and axilla (**A**) before treatment in comparison to the *left* breast and axilla (**B**) following 6 rounds of brentuximab vedotin and cyclophosphamide, doxorubicin, and prednisone chemotherapy.

Punch biopsy of the left breast revealed an acanthotic epidermis associated with a very dense and diffuse pan-dermal mononuclear infiltrate (Fig 2, *A*). Higher power examination revealed highly atypical mononuclear cells with anaplastic morphology and enlarged hyperchromatic nuclei and nucleoli (Fig 2, *B*) within the dermis and lumen of a blood vessel (Fig 2, *C*). Tumor cells demonstrated a CD4-positive, CD3-negative immunophenotype with a CD4:CD8 ratio >10:1. Strong and diffuse CD30, anaplastic lymphoma kinase (ALK), epithelial membrane antigen staining was appreciated in all mononuclear cells within the dermis (Fig 3, *A*-*C*). CD20, CD56, pankeratin, cytokeratin (CK) 7 (CK7), CK5/6, and CDX2 were all negative. Serology at the time of diagnosis demonstrated mild leukocytosis and thrombocytosis and normal lactate dehydrogenase levels. Flow cytometry of peripheral blood also revealed an increased CD4:CD8 ratio (12:1) and cytology demonstrated rare medium sized lymphocytes with atypical nuclear features. Positron emission tomography/computed tomography imaging (Fig 4, *A*) demonstrated marked skin thickening in the left breast area, left cervical, supraclavicular and axillary adenopathy (standardized uptake value 19.86) and enlarged lymph nodes in the deep subcutaneous soft tissues of the left anterior chest and right axilla (standardized uptake value >15). Focal areas of activity involving the T9 vertebral body were also appreciated (standardized uptake value = 4.25), consistent with involvement by the underlying ALCL. Breast mammography was negative for breast cancer. Given the presence of lymph node, peripheral blood and bone involvement at the time of workup and staging and positive ALK and epithelial membrane antigen immunostaining on histology, a diagnosis of secondary cutaneous ALCL was established.

Treatment was initiated with a combination of brentuximab vedotin and cyclophosphamide, doxorubicin, and prednisone chemotherapy for 6 cycles. Reported side effects included fatigue, diarrhea, mild weight loss and neuropathy. Clinical improvement of cutaneous manifestations occurred within 1 cycle of treatment. Repeat positron emission tomography/computed tomography scans after 3 and 6 cycles showed complete remission of disease (Fig 4, *B*). Complete clearance of cutaneous findings has been maintained for at least 12 months after treatment (Fig 1).

## Discussion

Here we describe the case of systemic-ALCL within an 80-year-old woman with no prior history of breast cancer or breast implants whose initial clinical presentation was a rash mimicking carcinoma en cuirasse of the breast. Carcinoma en cuirasse refers to the appearance of firm and indurated nodules and plaques developing on the breast that resemble a “cuirass” (or body shield). Although classically described in association with metastatic breast cancer,^6^ this phenomenon has only been described to occur secondary to lymphoma in a limited collection of cases (and termed lymphoma en cuirasse).^7^^,^^8^ To our knowledge, there have not been other previously reported definitive cases demonstrating lymphoma en cuirasse as a presenting cutaneous manifestation in ALCL.

PC-ALCL and secondary cutaneous ALCL can have histologic and immunophenotypic overlap but greatly vary with respect to prognosis and management; therefore, careful clinical evaluation with workup and staging including laboratory and imaging studies, and in some cases bone marrow biopsy is warranted. By definition, PC-ALCL should not have extracutaneous disease at the time of diagnosis but in some cases may have mild regional lymph node involvement without systemic disease. In contrast, as observed in this patient, secondary cutaneous ALCL may present with lymphadenopathy, blood and bone involvement. Although PC-ALCL and secondary cutaneous ALCL histologically exhibit diffuse dermal infiltrate composed of CD30^+^ tumor cells with anaplastic morphology, diffuse cytoplasmic ALK and epithelial membrane antigenpositive immunostaining is highly suggestive of secondary cutaneous systemic-ALCL rather than PC-ALCL (for review see^9^). Constitutive ALK-expression within systemic-ALCL has more commonly been described to be driven by the nucleophosmin-ALK translocation that histologically results in ALK expression within the nucleus and/or cytoplasm.^9^ Alternative ALK translocations have also been described in systemic-ALCL (namely *TRAF1-ALK*, *ATIC-ALK*, and *TMP-ALK*) that more frequently results in predominant cytoplasmic ALK expression.^9^ Consideration of the spatial localization of ALK expression can aid in further understanding the genetic underpinnings of the tumor microenvironment in the diagnostic workup of ALCL.

PC-ALCL has a favorable prognosis in localized disease with an overall 5-year survival of greater than 90%.^10^ Secondary ALCL has a more aggressive clinical course, worse prognosis and require multiagent chemotherapy regimens including brentuximab vedotin, a monoclonal antibody treatment that targets malignant T cells expressing CD30.^10^ Given systemic nature of the disease, treatment was undertaken with 6 rounds of brentuximab vedotin and cyclophosphamide, doxorubicin, and prednisone chemotherapy, resulting in complete remission maintained for 12 months thus far. In conclusion, we report a patient who presented with cutaneous lesions on the breast mimicking carcinoma en cuirasse and was subsequently diagnosed with secondary cutaneous ALCL. To our knowledge, such an unusual clinical presentation has not been previously described in cutaneous involvement by ALCL.

## Conflicts of interest

None disclosed.
